# Supplementary material for: Spray Dried Levodopa-Doped Powder Potentially for Intranasal Delivery
Source: Pharmaceutics. 2022 Jun 30;14(7):1384. doi: 10.3390/pharmaceutics14071384 (PMC9322363; doi:10.3390/pharmaceutics14071384)
Supplement: Supplementary file 1 [file pharmaceutics-14-01384-s001.zip › pharmaceutics-1781723-supplementary.pdf]

pharmaceutics

Supplementary Materials: Spray Dried Levodopa-doped Powder Potentially for Intranasal Delivery

Xuan Liu, Shen Yan, Mengyuan Li, Shengyu Zhang, Gang Guo, Quanyi Yin, Zhenbo Tong, Xiao Dong Chen and Winston Duo Wu

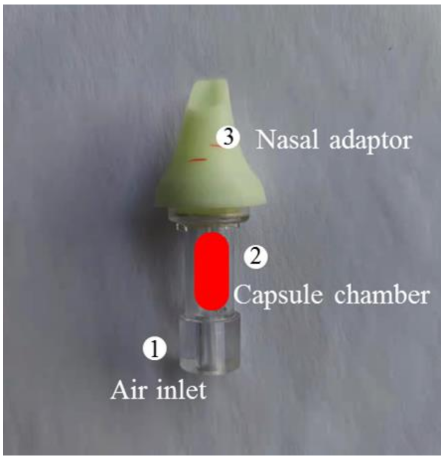

Figure S1. A simplified 3D-printed nasal powder delivery device to simulate the breath-powered Bi-Directional™ device 1-air inlet, 2-capsule chamber, 3-nasal adaptor.

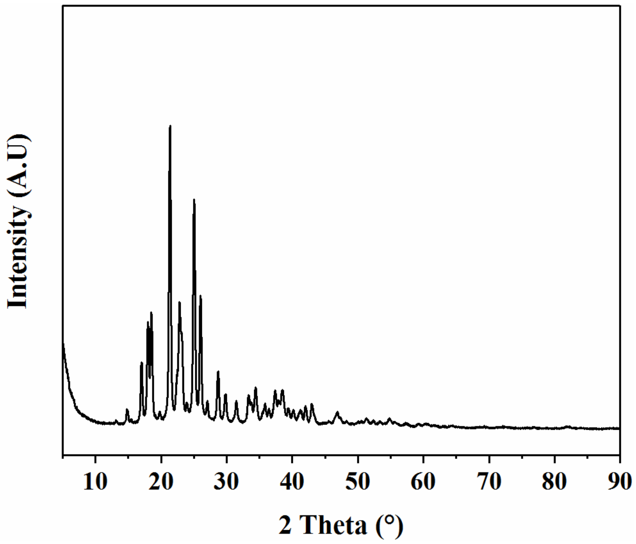

Figure S2. XRD patterns of the SD-L-dopa after storage.

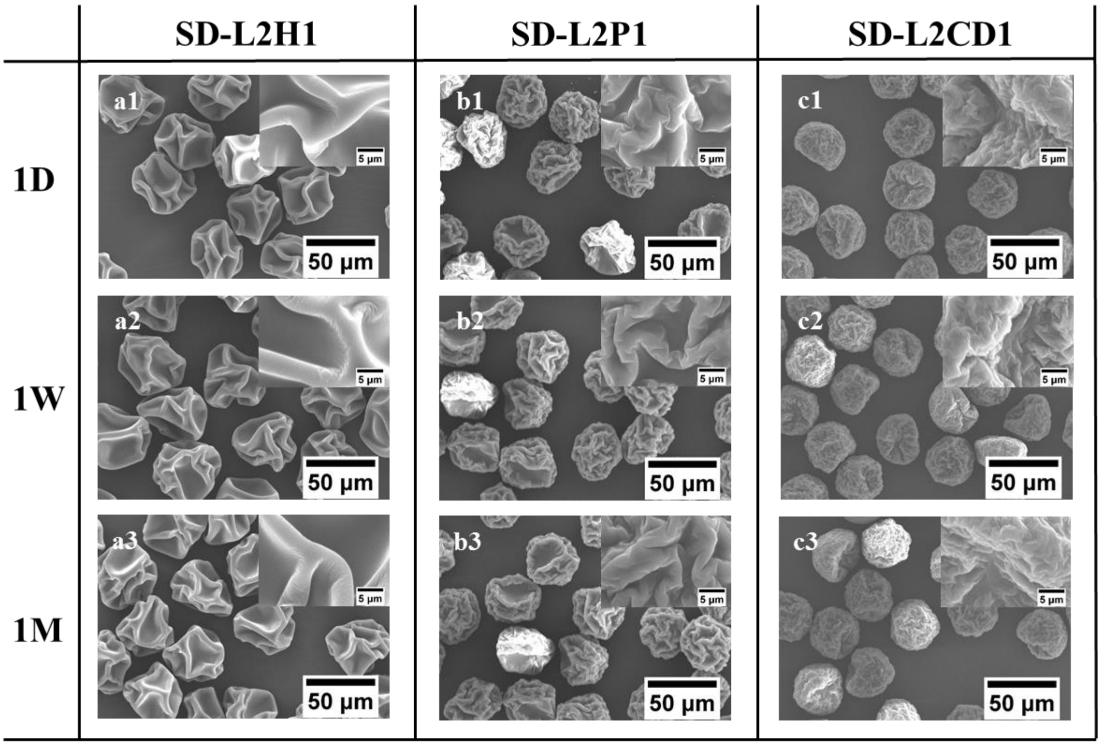

Figure S3. SEM images of SD samples after storage for different time: SD-L2H1 (**a1-3**); SD-L2P1 (**b1-3**) and SD-L2CD1 (**c1-3**) under the storage condition of 22 °C/18% RH for one day (1**D, a1, b1 and c1**), one week (1**W, a2, b2 and c2**) and one month (1**M, a3, b3 and c3**), respectively.

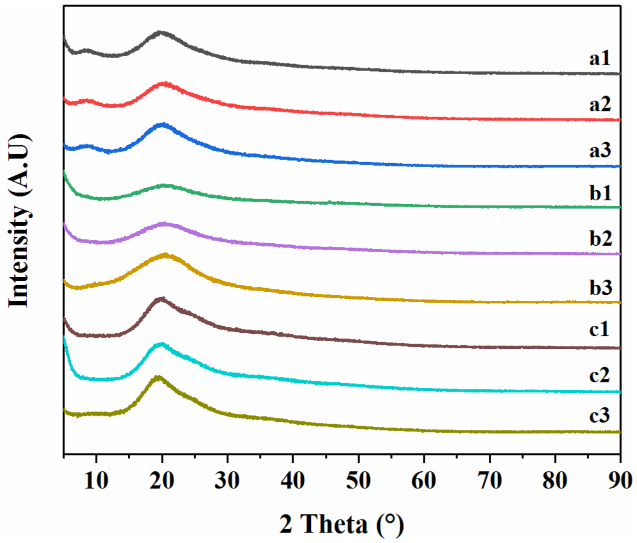

Figure S4. XRD patterns of SD samples after storage for different time: SD-L2H1 (**a1-3**); SD-L2P1 (**b1-3**) and SD-L2CD1 (**c1-3**) under the storage condition of 22 °C/18% RH for one day (**a1, b1 and c1**), one week (**a2, b2 and c2**) and one month (**a3, b3 and c3**), respectively.

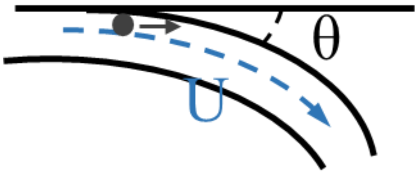

Figure S5. Schematic diagram of the inertial impaction process of particles.

Table S1. Emitted fraction and recovery fraction of SD-L1H2, -L1P2 and -L1CD2.

Scheme	Emitted Fraction (Total Dose %)	Recovery Fraction (Total Dose %)
SD-L1H2	99.33 ± 0.26	92.80 ± 6.01
SD-L1P2	99.38 ± 0.23	88.24 ± 2.04
SD-L1CD2	99.50 ± 0.02	90.56 ± 5.43
